# Supplementary figures and images for: Differences in Alternative Splicing between Yellow and Black-Seeded Rapeseed
Source: Plants (Basel). 2020 Jul 31;9(8):977. doi: 10.3390/plants9080977 (PMC7465011; doi:10.3390/plants9080977)

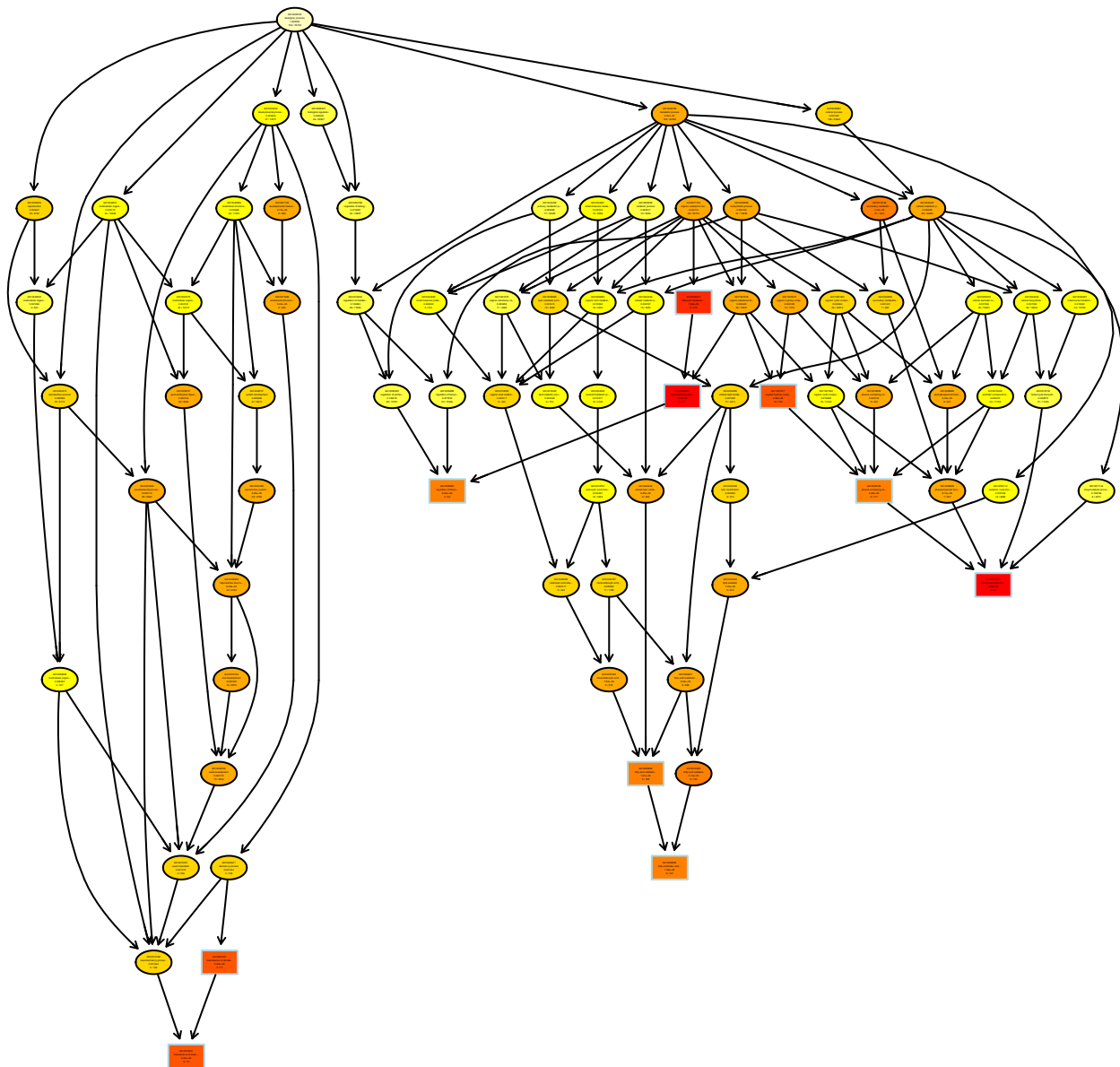

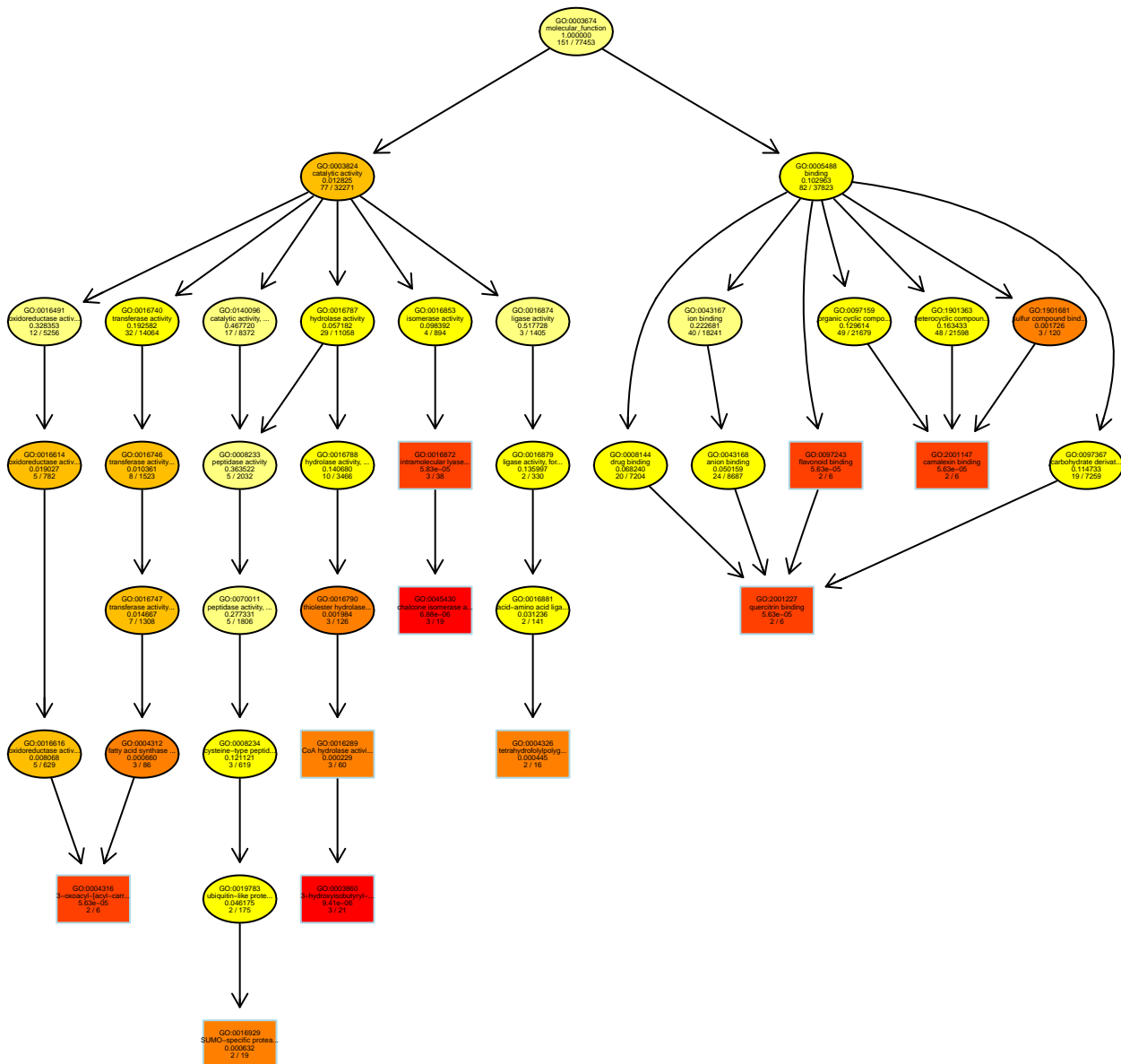

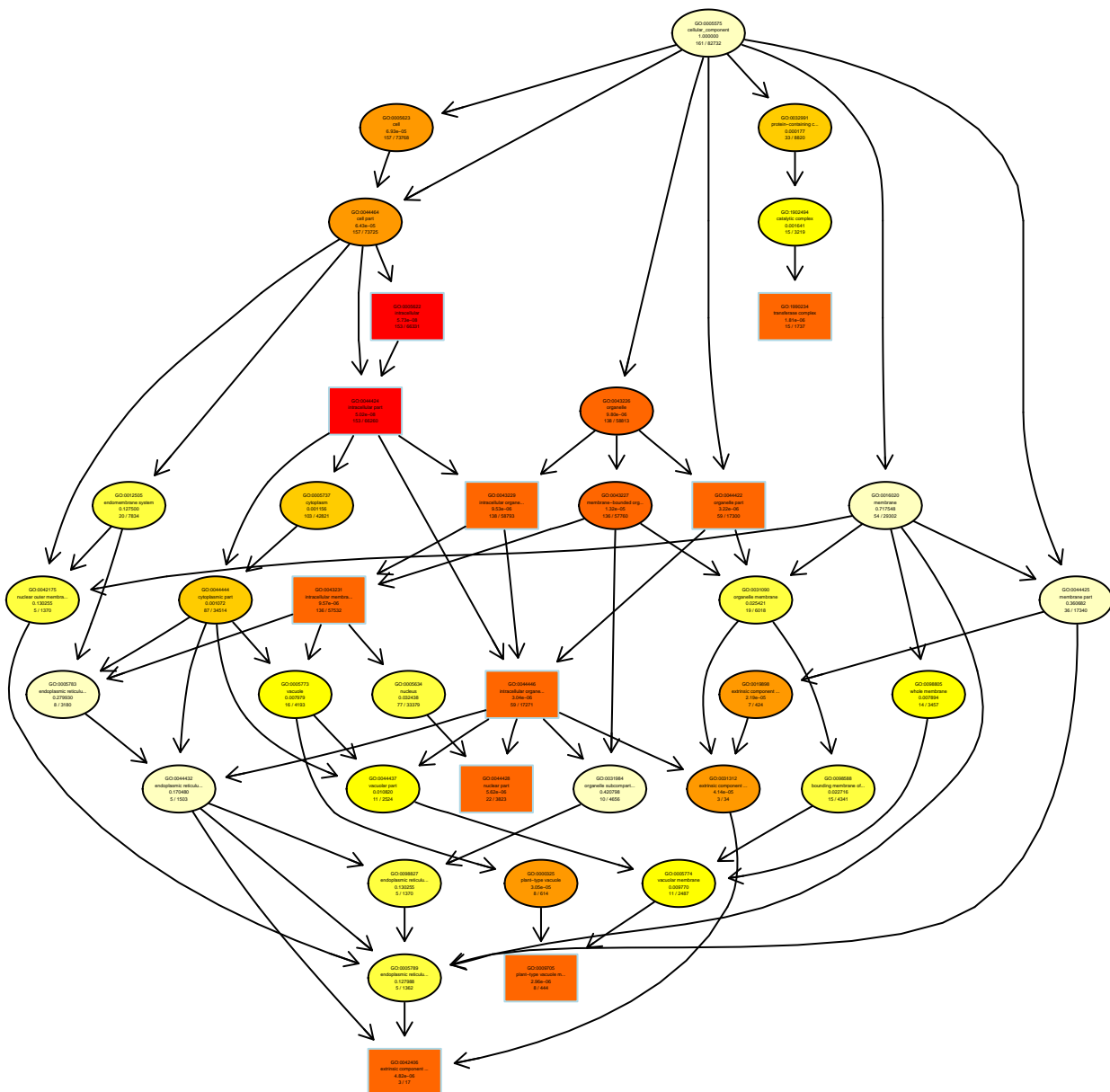

Supplement: Supplementary file 1 [file plants-09-00977-s001.zip › Supplementary Materials/Figure S1/CytoNodeblack.tab-go.pdf]

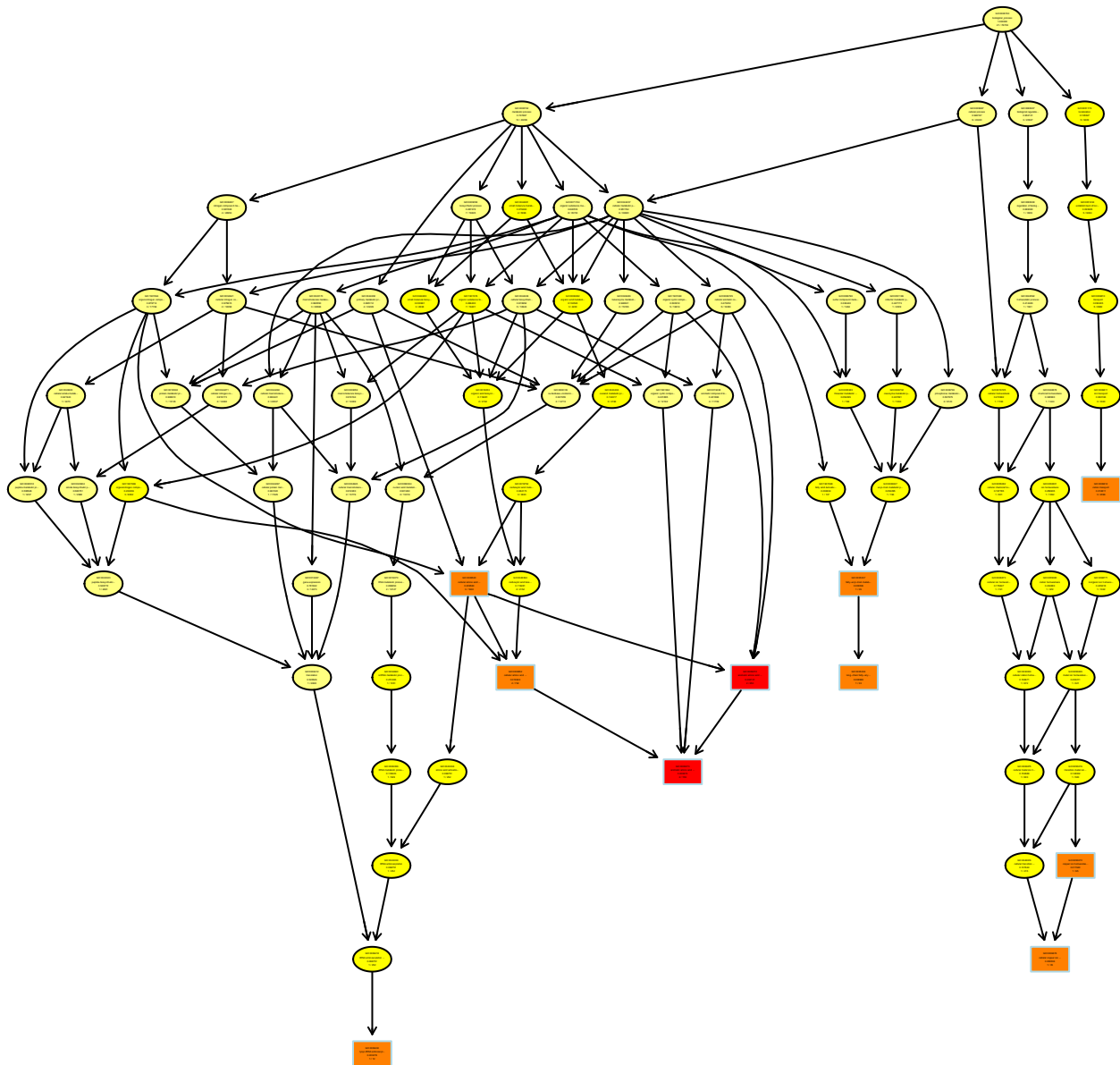

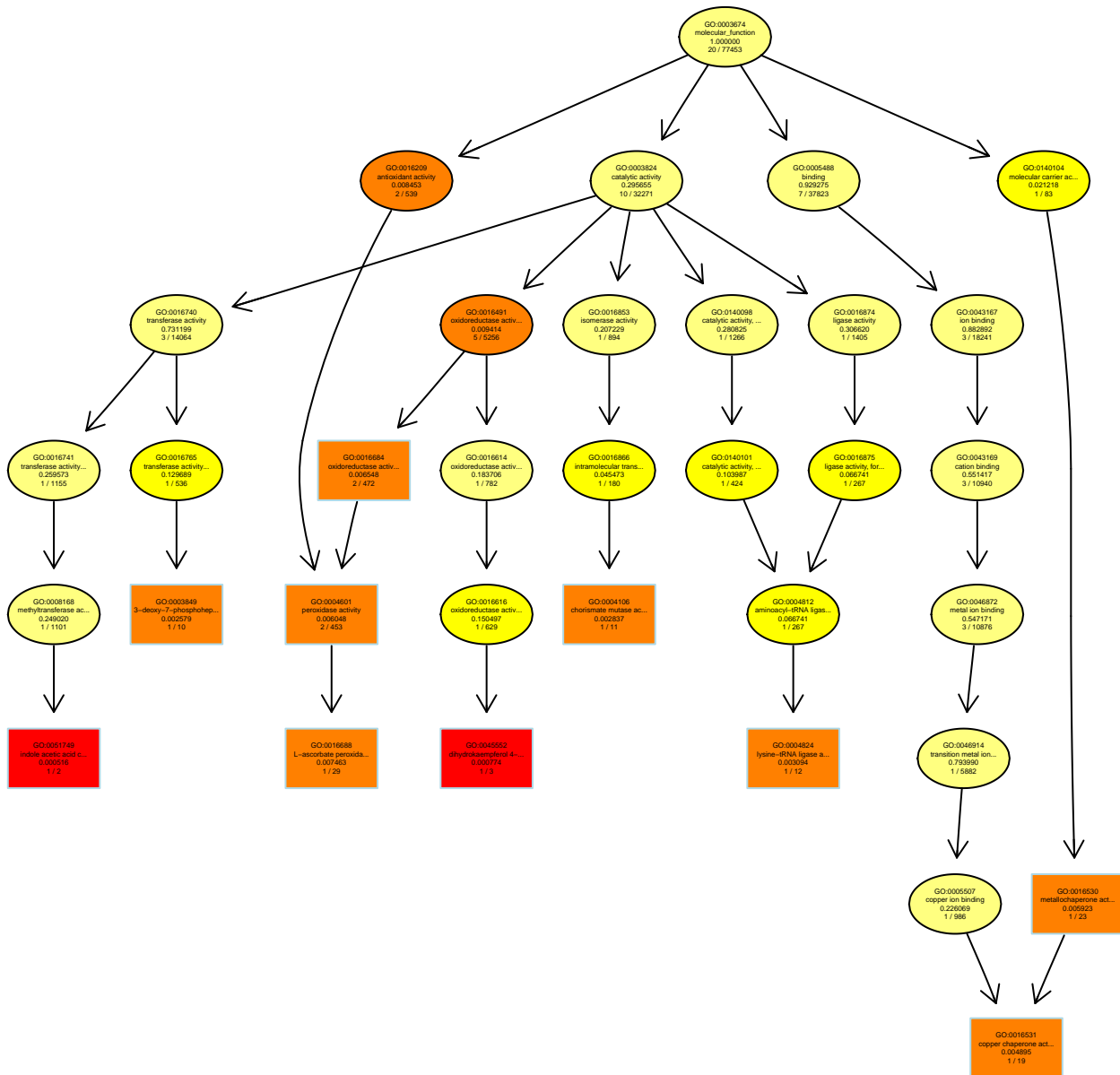

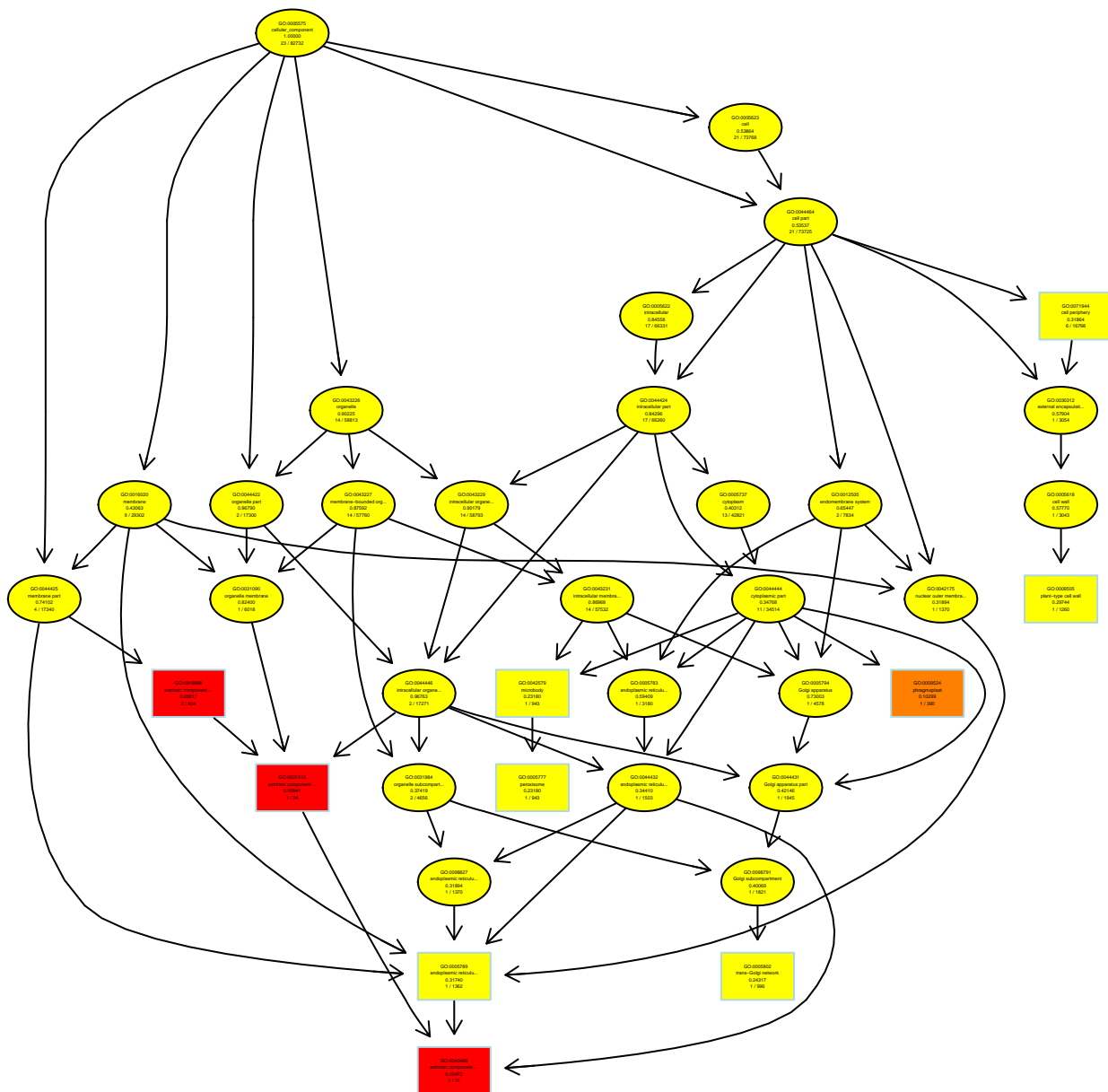

Supplement: Supplementary file 1 [file plants-09-00977-s001.zip › Supplementary Materials/Figure S1/CytoNodedarkgrey.tab-go.pdf]
